# Comparison of high and low stunting reduction groups using IFE-EFE matrix analysis in Central Java Province, Indonesia

**DOI:** 10.3389/fpubh.2023.1191473

**Published:** 2023-11-17

**Authors:** Mohamad Samsudin, Pramesthi Widya Hapsari, Martha Irene Kartasurya, Ahmad Syauqy, Muflihah Isnawati, Erna Kusuma Wati, Yusi Dwi Nurcahyani, Noviati Fuada, Suyatno Suyatno, Julian Dewantiningrum, Sunarto Sunarto, Nuryanto Nuryanto

**Affiliations:** ^1^National Research and Innovation Agency, Cibinong-Bogor, Indonesia; ^2^Faculty of Health, Jenderal Soedirman University, Purwokerto, Indonesia; ^3^Faculty of Public Health, Diponegoro University, Semarang, Indonesia; ^4^Faculty of Medicine, Diponegoro University, Semarang, Indonesia; ^5^Poltekkes Kemenkes Semarang, Semarang, Indonesia; ^6^Health Research and Development Center, Magelang, Indonesia

**Keywords:** stunting, factor analysis, internal factor evaluation, external factor evaluation, SWOT analysis, qualitative research stunting, qualitative research

## Abstract

**Background:**

The results of the 2021 Indonesian Nutritional Status Study (Studi Status Gizi Indonesia, SSGI) showed a 6.8% declining prevalence of stunting in Central Java from 2019 to 2021. However, the prevalence decreases in the regency level of Central Java varied from 0.1 to 20.3%. This study aimed to analyze the external and internal factors that influenced the stunting reduction prevalence in Central Java Province, Indonesia.

**Methods:**

This qualitative study was conducted over 2 months, from April to May 2022. In-depth interviews were used to explore the supporting factors (external, internal, and strategies) and the challenges (internal, external, and solutions) in implementing stunting reduction acceleration programs. The supporting factors and challenges were compared between the groups with high Gro, Sra, Kla, and Pek (GSKP) and low Sur, Mag, Teg, and Pat (SMTP) reduction prevalence. Data were analyzed using internal factor evaluation (IFE) and external factor evaluation (EFE).

**Results:**

In quadrant II, the high-reduction group (GSKP) had IFE and EFE scores of 2.61 and 2.76, respectively. In quadrant IV, the low-reduction group (SMTP) had IFE and EFE scores of 1.86 and 1.62, respectively. The high-reduction group (GSKP) was better than the low-reduction group (SMTP) in using external opportunities and internal strengths by avoiding external threats and minimizing the weakness in the stunting reduction acceleration program in each area. The existence of superior programs and innovations were the strengths that differentiated the high and low groups. Conversely, the low-stunting reduction group struggled to overcome major challenges, especially lacking leadership capacity. From the outside, lack of program sustainability at the village level and budget restraint were the threats found in both groups.

**Conclusion:**

Compared with the low-stunting reduction group, the high group could maximize their strengths and use the opportunities to implement stunting reduction programs.

## 1 Introduction

A national data survey in Indonesia showed that the prevalence of stunting declined from 37.2% in 2013 to 24.4% in 2021 (1–4). It means the prevalence reduction in 8 years was 1.6% per year on average. In the last years, stunting prevalence declined from 27.7% in 2019 to 24.4% in 2021 (3.3% or 1.65% per year). Although the stunting prevalence in Indonesia has decreased, it still does not meet the 2024 national target (Indonesian Medium-Term Development Plan), which is 14%. This indicates that stunting prevalence needs to decrease by approximately 10.4 or 3.5% per year. Therefore, it is necessary to implement an appropriate strategy to reduce the prevalence of stunting in order to achieve the national target by 2024 (5).

Social determinant was the most important factor that affected stunting reduction. Multisectoral and community approaches, political commitments, increasing the national budget, and interventions focused on low-income communities supported the decline of stunting (6–8). The Indonesian Government set the action-plan recommendations on stunting reduction to increase convergence, coordination, consolidation, and expanding the program scope (9). In addition, based on the National Medium Term Development Plan, the national strategy for stunting reduction was emphasized in President Regulation No 72/2021 about Stunting Reduction Acceleration (5).

The 2019 and 2021 Indonesian Nutritional Status Study (Studi Status Gizi Indonesia, SSGI) showed the disparity and diversity of stunting reduction of the province. The results also showed that some provinces had greater reductions in stunting prevalence than the national reduction. One of them is Central Java Province. The prevalence reduction in Central Java was greater than the national prevalence, which was 6.8%, from 27.7% in 2019 to 20.9% in 2021 (1). Moreover, Central Java had the seventh lowest stunting prevalence in Indonesia. Central Java Province was also rewarded as the implementor in fostering and supervising eight integrated stunting reduction convergence actions in 2020 (1). However, the stunting reduction prevalence among the regencies and cities in Central Java was strikingly varied. Stunting prevalence reduction from 2019 to 2021 showed a high variation from only 0.1% in Magelang District to a 20.3% reduction in Grobogan District (1, 3, 5, 9). To accelerate the prevalence of stunting reduction, the Central Java Government rewarded the district with the best performance in implementing the eight convergence stunting programs (10).

Nevertheless, every district surely experienced difficulty in the implementation. Ego sectoral in the local government agency was one of the internal challenges as socialization and commitment were still lacking. Furthermore, the programs had no clear guidelines (technical and implementation instructions). Information sharing was sometimes interrupted and cut off (11). Strengths, weaknesses, opportunities, and threats (SWOT) analysis is an instrument to analyze the factors that influence the performance of a program or business (12, 13). In the healthcare program, the SWOT analysis is also used in the early stage of intervention or evaluation of the program. Three studies on stunting programs that used the SWOT analysis were mainly conducted at the district level and used a qualitative approach (14–16). One study used the SWOT analysis in the early stage of intervention (14). Two studies used the SWOT analysis to evaluate the stunting program at the district level (15, 16). One of the two studies at the district level was not only using a qualitative approach but also using the internal factor evaluation (IFE) and the external factor evaluation (EFE) analyses (15). The IFE and EFE analyses are widely known as strategic instruments to analyze organizational and environmental factors affecting a company and identify the most appropriate strategy. This model has been shown to improve the business strategy at a more comprehensive corporate level (13). Therefore, using the IFE and EFE analyses could be an appropriate instrument to capture the implementation of the stunting reduction program in Central Java Province. Moreover, the IFE and EFE analyses could give information on which matters the most in Central Java Province and the improvement strategy that could be carried out.

In order to learn a lesson from the implementation of the eight convergence stunting programs, this study aimed to explore and analyze the external and internal factors affecting the achievements of the stunting reduction at the city/regency level. Comparing strengths, weaknesses, opportunities, and threats between districts with high- and low-stunting reduction could recommend the acceleration of stunting reduction programs.

## 2 Materials and methods

### 2.1 Population

Data collection in this qualitative study was done from April to May 2022 in Central Java Province. Central Java has the highest population density in Indonesia (17). Moreover, Central Java is also the most expansive province on Java Island. Therefore, Central Java represents Indonesian characteristics, providing a good overview of Indonesia, a developing country. A qualitative study was done primarily using in-depth interviews. The study used in-depth interviews to explore supporting factors (external, internal, and strategies) and the challenges (internal, external, and solutions) in implementing the stunting reduction acceleration programs. This study also reviewed the results of the nutrition programs. A comparison of the supporting factors and challenges was done between two groups, the high-stunting reduction prevalence (GSKP), and the low-stunting reduction prevalence (SMTP).

### 2.2 Data collection

The informants were the officials of the Local Development Agency Offices, who were related to the stunting reduction programs in the eight districts. In-depth interviews were used to explore perspectives, personal feelings, and experiences, particularly during the implementation of the stunting reduction program at the districts. There were 36 informants selected purposively according to their responsibility in the stunting reduction program. The informants were from the District Health Office; the Community and Village Empowerment Office; the Women Empowerment, Child Protection, and Family Planning Office; the Social Affair Office; the Agricultural Office; and the Public Works and Public Housing Office (Water Supply, Hygiene, and Sanitation Program). The researchers interviewed the informants at the Local Development Agency office using interview guidelines based on a national strategy to accelerate stunting reduction and procedures for implementing integrated stunting reduction interventions in districts. The interviews lasted for 1–2 h and were recorded. During the interviews, documents related to the stunting programs were also checked for verification. The documents included the regional planning documents, the public health profiles, the regulations, and the reports related to the stunting programs.

### 2.3 Data analysis

The IFE and EFE matrices were known as a method to analyze strengths and weakness as internal factors as well as opportunities and threats as external factors in business strategy. The use of the IFE and EFE matrices in analyzing the comparison between high and low stunting reduction groups at Central Java Province could give more overview of supporting factors (strength, opportunities, and strategies) and the inhibiting factors (weakness, threats, and solutions) in the implementation of the stunting reduction program (15, 18).

The first stage of the IFE and EFE analyses was identifying the strengths and weaknesses as the internal factors, and the opportunities and threats as the external factors from in-depth interview transcripts. Using three-level coding, information in the transcripts was categorized into strengths, weaknesses, opportunities, and threats (SWOT) in implementing the district-level stunting reduction programs. The identification of SWOT was discussed among the research team until an agreement was reached.

The second step was the weighting of the strategic factor components. The weighting was determined by comparing each factor on a scale of 0 to 1. The experts discussed the weighting of the factors based on guidelines for implementing integrated stunting reduction interventions in districts until they reached a consensus.

The third step was determining the rating of each factor. The experts rated the factors on a scale of 1–4. In the IFE, a score of 1, 2, 3, and 4 indicates a large weakness, a small weakness, a little strength, and a great strength of each component, respectively. However, in the EFE, a score of 1, 2, 3, and 4 indicates below the average response, an average response, above the average response, and a very good response, respectively. The fourth step was calculating the value of each factor by multiplying the rating and weight and then summing them up (15, 18).

A total IFE value of less than 2.5 indicated a weak internal position of the institution, while a total value above 2.5 indicated a strong internal position of the institution (15). The total EFE value of 1.0 means that the institution cannot take advantage of the opportunities or has not minimized the external threats. A total EFE score of 4.0 indicated that the institution’s response is excellent in seizing the opportunities and avoiding the existing threats (15). This study was conducted according to the guidelines of the Declaration of Helsinki and approved by the Health Research Ethics Committee of the Public Health Faculty of Diponegoro University (No. 134/EA/KEPK-FKM/2022).

## 3 Results

The results of the analysis on supporting (internal and external) and inhibiting factors (internal and external) for accelerating stunting reduction in eight districts in Central Java Province were explained in IFE and EFE matrices. Evaluation of internal factors includes the strengths and weaknesses, while evaluation of external factors includes the opportunities and threats.

Table 1 shows the IFE matrix comparison between the high- and low-stunting prevalence reduction groups. The values of the IFE matrix indicated a strong internal position of the institution. The score value of the IFE matrix in the high-reduction group (2.61) was higher than the low-reduction group (1.86). The main strengths that support the acceleration of stunting reduction in the high group were support for regional policies and regulations (0.225), strong commitment from the leaders (0.211), priority programs or innovations (0.197), and good coordination (0.197). The main strengths that support the acceleration of stunting reduction in the low group were adequate budget (0.180), support for regional policies and regulations (0.169), good coordination (0.169), and strong commitment from the leaders (0.154).

**Table 1:** The IFE matrix according to high- and low-stunting reduction groups.

Internal factors (S-W)	Weight	High-stunting reduction group		Low-stunting reduction group	
		Rating	Score value	Rating	Score value
Strength					
Strong commitment from leaders	0.06	4	0.211	3	0.154
Support for regional policies and regulations	0.06	4	0.225	3	0.169
Having priority programs or innovations	0.06	4	0.197	2	0.094
Good coordination	0.06	4	0.197	3	0.169
Good human resources (quantity and quality)	0.04	3	0.135	3	0.135
Adequate budget	0.04	4	0.180	4	0.180
Institutional strengthening at the district level	0.04	4	0.180		0.000
Synchronization of stunting management programs at the rural level by strengthening the rural institution	0.04	4	0.180		0.000
The process of monitoring and evaluation is already running well	0.04	3	0.135	3	0.135
Application-based data are available and integrated	0.04	3	0.150	3	0.135
Adequate tools (e.g., anthropometric measurements)	0.03	3	0.101		0.000
Regular formal and informal meetings	0.03	3	0.101	3	0.101
New programs from the government	0.02	3	0.067		0.000
The multimedia is active for coordination	0.01	3	0.034		0.000
Weakness					
Limited budget	0.06	1	0.056	1	0.056
Lack of coordination	0.06	1	0.056	1	0.056
Lack of commitment	0.06	1	0.056	2	0.112
Lack of data availability and quality	0.04	1	0.045	1	0.045
Lack of human resources	0.04	1	0.045	2	0.090
Lack of cadres	0.03	2	0.067	2	0.067
Inadequate tools	0.03	1	0.034	2	0.051
Low education level	0.03	2	0.067	2	0.067
Lack of socialization and training of the government officers	0.02	2	0.045		0.000
Policies from the Center are less regionally adapted; the replacement of the person in charge of the program	0.01	2	0.022	2	0.022
Technical constraints in the implementation: non-cash food subsidy distribution, optimization of sustainable food house area	0.01	2	0.022	2	0.022
Total	1.00		2.608		1.860

The main weaknesses that counter the acceleration of stunting reduction in the high group were low education level (0.067), lack of cadres (0.067), limited budget (0.056), lack of coordination (0.056), and lack of commitment (0.056). The main weaknesses that counter the acceleration of stunting reduction in the low group were lack of commitment (0.112), lack of human resources (0.090), low education level (0.067), and lack of cadres (0.067).

Table 2 shows the EFE matrix on the high- and low-stunting reduction groups. The values of the EFE matrix indicated a strong internal position of the institution. The value of the EFE matrix in the high-stunting reduction group (2.755) was higher than in the low-stunting reduction group (1.618). It showed that compared with the low group, the high-stunting reduction group could take advantage of the strengths and minimize the weaknesses in the internal program.

**Table 2:** The EFE matrix in high- and low-stunting reduction groups.

External factors (O-T)	Weight	High-stunting reduction group		Low-stunting reduction group	
		Rating	Score value	Rating	Score value
Opportunities					
Availability of guidelines and policies	0.12	4	0.471		0.000
Availability of budget	0.12	4	0.431	3	0.353
Support from non-government organizations (NGOs), academicians, and the media	0.12	4	0.471	3	0.353
Community empowerment	0.09	3	0.265	3	0.265
Threats					
Low sustainability of the program	0.15	2	0.294	1	0.147
COVID-19 pandemic	0.12	2	0.235	1	0.118
Changes in authority and policies	0.12	2	0.235	2	0.235
No support from NGOs and stakeholders	0.09	2	0.176	1	0.088
Low motivation and awareness	0.06	2	0.118	1	0.059
Lack of clean water sources due to natural conditions	0.03	2	0.059		0.000
Total	1.00		2.755		1.618

The main opportunities that support the acceleration of stunting reduction in the high group were the availability of guidelines and policies (0.471), support from non-government organizations (NGOs), academicians, and the media (0.471), and budget availability (0.431). The main opportunities that support the acceleration of stunting reduction in the low-reduction group were budget availability (0.353) and support from non-government organizations (NGOs), academicians, and the media (0.353).

The main threats that counter the acceleration of stunting reduction in the high-reduction group were the low sustainability of the program (0.294), the COVID-19 pandemic (0.235), and changes in authority and policies (0.235). The main threats that counter the acceleration of stunting reduction in the low-reduction group were organizational dynamics: changing authority issue, stunting regulation team, the appointment of a secretariat (0.235), and no support from NGOs and stakeholders (0.088).

**Figure 1:**
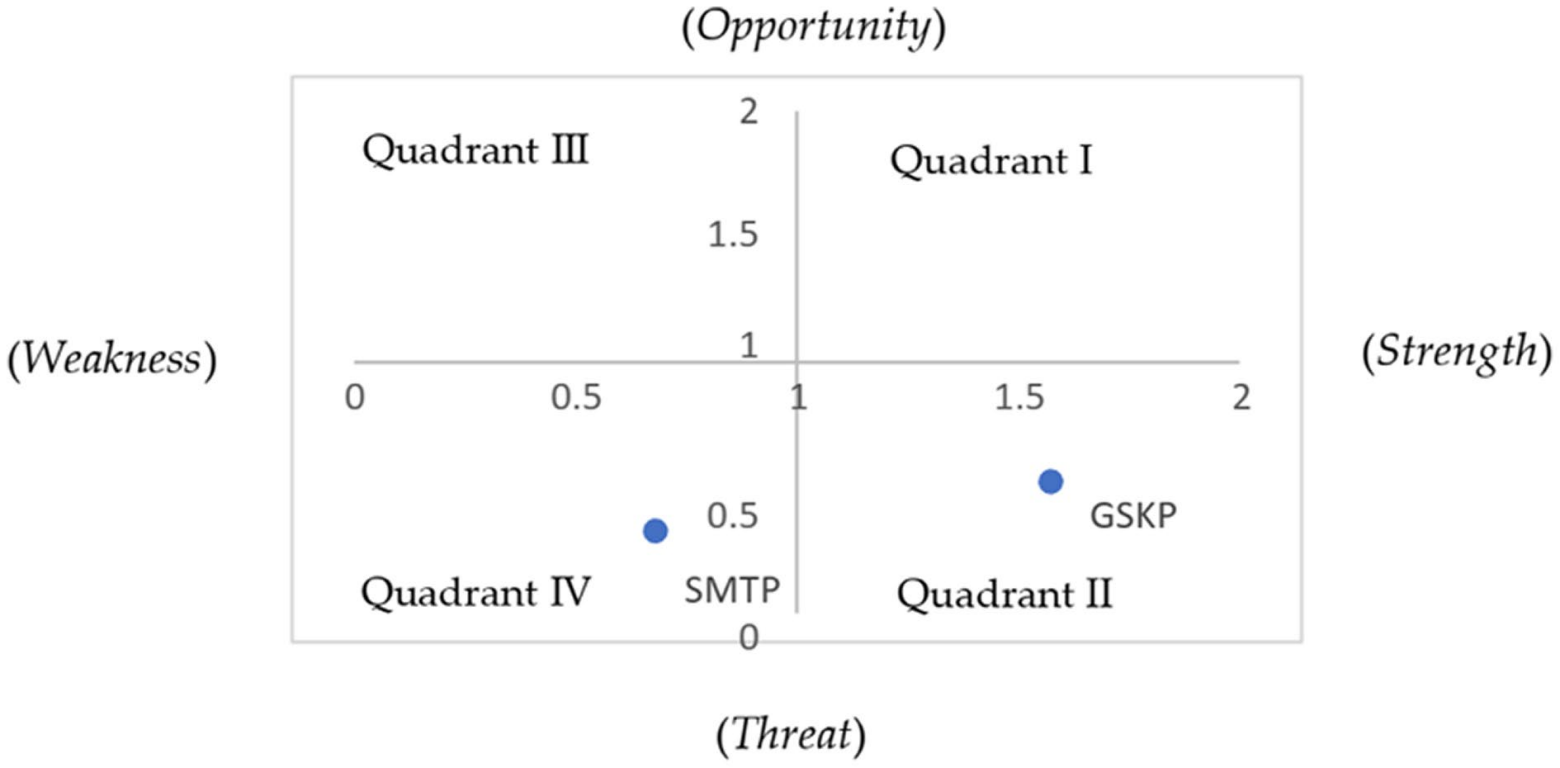
SWOT analysis.

The results of the SWOT analysis in this study that compared the internal factors (strengths and weaknesses) and external factors (opportunities and threats) indicated that the high-reduction group was in quadrant II (interaction between strengths and threats). It is noted that the supporting components in stunting reduction were vital, although they experienced threats. In contrast, the low-stunting reduction group was in quadrant IV (interaction between weaknesses and threats). In this condition, the low-stunting reduction group experienced difficulties in managing the weaknesses while overcoming the challenges (Figure 1).

## 4 Discussion

### 4.1 The internal factor evaluation

The study compared the internal and external factors that support the acceleration of stunting reduction between high and low groups and found similarities and differences in each aspect. In the high group, the strengths were supporting regional policies and regulations, strong commitment from the leaders, having priority programs or innovations, and good coordination. Meanwhile, in the low group, the strengths were adequate budget, support for regional policies and regulations, good coordination, and strong commitment from the leaders. The weaknesses in the high group were the low education level of the community, lack of cadres, limited budget, lack of coordination, and lack of commitment. The main weaknesses that counter the acceleration of stunting reduction in the low group were lack of commitment, lack of human resources at the district level, low education level of the community, and lack of cadres.

The strengths found in both groups were the strong commitment from the leaders and support for regional policies and regulations. However, the low-reduction group had a lower score (rating 3) than the high-reduction group. It could be due to no consensus on resolving nutritional issues, such as malnutrition, and the lack of awareness from families and communities. A lack of leaders’ commitment to cope with nutritional problems leads to insufficient nutritional investment, resulting in less impactful programs and reinforcing the lack of commitment (19). Several countries reported that consistencies and political commitments to budget and program implementation are essential factors that influence the success of nutrition improvement programs. The political commitment included raising funds and inviting advocates from national and international institutions, NGOs, universities, and others (10).

The existence of special programs and innovations were found to be strengths in the high group, but not in the low group. Excellent programs and innovations are more prominent and were created by the high-reduction group, with a 4 rating, than by the low-reduction group, with a 2 rating. Innovations conducted by the four regencies in the high-reduction group were integrating the monitoring efforts to reduce stunting prevalence through a messenger application and activities that used local cultures and wisdom, such as eating together and using local food. Local policies, outstanding programs, and innovation have massive rules supporting stunting prevention. The innovations are the retrieval process, which can be done by increasing the resources and expanding the target access (20).

A similar weakness found in both groups was the lack of human resources. Although in the high-reduction group, the issues of human resources were related to the health cadre’s capacities, the problems in the low-reduction group were the lack of implementor staff at the district to village level and the low education level of the communities. The lack of leadership capacity was an issue of human resources in the low-reduction group. Furthermore, low public awareness was mainly a weakness in the high-reduction group.

### 4.2 The external factor evaluation

The main opportunities found similar in both groups were the budget availability and support from non-government organizations (NGOs), academicians, and the media. Budget availability became a main opportunity for supporting the stunting reduction program. Since the acceleration program was launched in 2018, the government has prepared the budget. It is one of the funding sources from the special allocation fund, allocated by the central government to the local ones, especially in the priority locations for stunting reduction. This budget availability also showed a strong commitment from the central government to prevent stunting among children under 5 years of age (10).

Support from private sectors, NGOs, and universities is crucial in preventing stunting. Private sectors and NGOs can contribute through nutritious food production and marketing. At the same time, academicians can carry out studies to find the best evidence-based programs or be informants in coordination or socialization meetings (21). The importance of multisectoral roles in solving the stunting/malnutrition problem must be highlighted, not just as a mere commitment seen in the high-reduction group. The results of the study on multisectoral intervention to accelerate the stunting reduction in nine Sub-Saharan African countries showed that it had a potentially decreasing number of stunting. During 1 year of program implementation, food security and diversity were increased. This condition declined stunting about 43%, more than the initiated number of children in the third year (22). Multisectoral approaches also succeeded in reducing the number of stunting in Peru, Bolivia, and India (23, 24). Synergy must prioritize financial and human resources for medical, education, basic infrastructure, and other spending relevant to stunting (9). Communication between the stakeholders and coordination of activities into one joint venture was also needed (25).

The availability of guidelines and policies was found only as an opportunity in the high group. The policies as the basis of program implementation came from the central government policies (ministry) and the local ones. Furthermore, policies and regulations must be followed at the regional to village level and involve the medical and related sectors. The implementation is in the form of strategic and work plans of the institutions related to stunting reduction. The practices of policies and nutrition programs are also evidence of commitment to nutrition management (26). In the low group, cadres and community awareness helped the low-reduction group’s local government to support the implementation program.

A strategy to build community participation, especially with limited access to healthcare, can contribute effectively to handling nutrition problems (27). With increasing public education and awareness of nutrition and stunting prevention, the government has regulated the national acceleration for nutrition improvement. It is the efforts of both the government and society to accelerate the nutrition improvement prioritized to the first 1,000 days of life.

The lack of a sustainable stunting reduction program was shown as a threat in both groups, mainly in the low-reduction group. Since the stunting program, innovation was not optimal and the community felt the burden for success, the program’s continuity was hard to achieve and caused a lack of motivation. Stunting prevention is a national strategic program conducted through related ministries and local governments. The program’s sustainability will determine its success in the stunting reduction program. The program’s sustainability depends on the community’s commitment and involvement. The combination of policies, cross-sectoral collaboration, community involvement, and the use of community-based applications are the characteristics of the program’s success. Even though there are program innovations, if there is no commitment and community involvement, the intervention objectives will not be achieved (28).

Lack of data quality and availability were identified in both groups, while appropriate and good quality data can support the success of a program. Data are required for compiling the program, activity planning, and decision-making. Data collected periodically can strengthen the capacity to provide nutritional care (29). The main problem is that the data have not been integrated. Integrated data are crucial to obtaining information on nutritional status and program performance, which are used to identify the nutrition problem and decide and determine the policies. Nationally available data are from the Electronic Community-Based Nutrition Recording and Reporting (e-PPGBM) application. The use of e-PPGBM data with quality improvements would be useful.

### 4.3 The internal factor evaluation and external factor evaluation matrix position

The SWOT analysis combining the IFE and EFE matrices showed that the high-stunting reduction group had better values than the low-stunting reduction group. The score value of the high-reduction group is in quadrant II. This Cell B position is an interaction between threat and power that sign a strong organization but faces big challenges. The high-reduction group also had a stronger internal position in the institution than the low-reduction group. In other words, the high-reduction group had a better capacity to organize the strengths and take advantage of the opportunities; thus, the challenges and threats could be resolved.

The examples of the strategy implemented were as follows: (1) The leader’s commitment and more detailed regulation support to sustain the program at the rural level as well as regular and leveled monitoring and evaluation; (2) support to the local policies with good operational and strong cross-sectoral cooperation to face the issue of changing authority; (3) increasing the excellent programs and innovations through the outside groups’ contributions (Penta helix); (4) maximizing the resources and exploring funding sources from other sides, such as CSR, NGOs, and others; (5) public empowerment to increase comprehension, ability, and willingness to prevent stunting; and (6) accelerating the fulfillment of clean water in high-stunting prevalence areas.

A diversification strategy was needed in the high group to face a severe challenge. It is suggested to expand the variety of tactical strategies. There must be a mobilization of the resources, such as the organization’s power, to tackle the outside threats and even change them into opportunities. Variations and innovations of organizational strategy must be added to accelerate the stunting reductions to achieve a stunting prevalence of 14% in 2024. If they rely only on the previous strategy, the prevalence of stunting will stay the same.

The score value of the low-stunting reduction group is in quadrant IV. This D-cell position is the meeting point between the organization’s weakness and outside threats that sign a weak condition and face large challenges. Therefore, an inappropriate decision will bring a massive problem to the organization. The chosen strategy is a survival strategy, meaning that the internal condition of an organization is in the dilemmatic options. The strategy that should be taken is controlling the damage and internal performance to prevent worse achievements. This should be maintained while constantly maintaining self-improvement. Another strategy that can be implemented is a weakness threat/damage control strategy (minimizing the weakness and hindering the threat), which is as follows: (1) building and increasing the leader’s commitment at all levels (regency, district, and village); (2) increasing awareness of the importance of coordination and cooperation among the cross-sectors by activating stunting prevention teams; (3) improving the management and quality of programs related to the efforts; and (4) having an ‘emergency plan’ to face external threats.

The result of the study provided an overview of the implementation of the stunting reduction program not just in Central Java, but also in other provinces with similar reductions. Other than that, the IFE and EFE matrices gave an insight into improving stunting reduction strategy in terms of local program innovation as the strength, human resources capacity as the weakness, and program sustainability as the threat to increasing stunting reduction at the national and province levels. Interviews with qualified informants resulted in accurate information about the implementation of a stunting reduction in Central Java. However, the interviews could not fully go through each of the intervention activities at a local government agency.

## 5 Conclusion

In summary, the evaluation of internal and external factors in the high-stunting reduction group showed an IFE of 2.61 and EFE scores of 2.76, respectively. Those scores are greater than those in the low-stunting reduction group, with IFE and EFE scores of 1.86 and 1.62, respectively. Analyzing IFE and EFE shows that the group of regencies/cities with high-stunting reduction has strong internal conditions by maximally using the remaining strengths and decreasing the weaknesses in reducing stunting in their areas. This group has used the existing opportunities and hindered the outside threat well. The high-reduction group is in the quadrant II position, and strategy diversification is needed. It also needs to expand the variety of tactical strategies. The effort to mobilize the resources is the organization’s power to tackle outside threats and even change them into opportunities. On the other hand, the group with the low-stunting reduction has a weak internal condition and massive outside threats. The score value of the low-stunting reduction group is in quadrant IV. The strategy chosen is a survival strategy in which they need to control the damage and internal performance so that the condition does not worsen.

## Data availability statement

The datasets presented in this article are not readily available because data are available from the authors upon reasonable request and with permission of the Ministry of Health. Requests to access the datasets should be directed to MS, sam.bp2gaki@gmail.com.

## Ethics statement

The studies involving humans were approved by The Health Research Ethics Committee of the Public Health Faculty of Diponegoro University (No. 134/EA/KEPK-FKM/2022). The studies were conducted in accordance with the local legislation and institutional requirements. The participants provided their written informed consent to participate in this study.

## Author contributions

MS, PWH, MIK, AS, MI, EKW, YDN, NF, SSuy, JD, SSun, and NN were responsible for the conception and design of the study. MS, PWH, MIK, and AS were responsible for managing and retrieving the data. MS and PWH performed the statistical analysis and interpretation of data. MS and PWH wrote the manuscript. AS performed a critical review. All authors contributed to the article and approved the submitted version.
